# ADAMTS-12: A Multifaced Metalloproteinase in Arthritis and Inflammation

**DOI:** 10.1155/2014/649718

**Published:** 2014-04-28

**Authors:** Jianlu Wei, Brendon Richbourgh, Tanghong Jia, Chuanju Liu

**Affiliations:** ^1^Department of Orthopaedic Surgery, New York University Medical Center, New York, NY 10003, USA; ^2^Department of Orthopaedic Surgery, Shandong University, Jinan, Shandong 250012, China; ^3^Department of Cell Biology, New York University School of Medicine, New York, NY 10016, USA

## Abstract

ADAMTS-12 is a member of a disintegrin and metalloproteinase with thrombospondin motifs (ADAMTS) family of proteases, which were known to play important roles in various biological and pathological processes, such as development, angiogenesis, inflammation, cancer, arthritis, and atherosclerosis. In this review, we briefly summarize the structural organization of ADAMTS-12; concentrate on the emerging role of ADAMTS-12 in several pathophysiological conditions, including intervertebral disc degeneration, tumorigenesis and angioinhibitory effects, pediatric stroke, gonad differentiation, trophoblast invasion, and genetic linkage to schizophrenia and asthma, with special focus on its role in arthritis and inflammation; and end with the perspective research of ADAMTS-12 and its potential as a promising diagnostic and therapeutic target in various kinds of diseases and conditions.

## 1. Introduction

**Figure 1:**
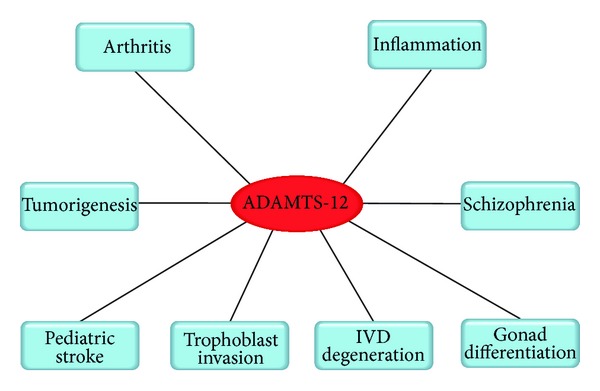
A schematic representation of ADAMTS-12's multiple functions.

A disintegrin and metalloproteinase with thrombospondin motifs (ADAMTS) family consists of a precisely ordered modular organization that includes at least one thrombospondin type I repeat [1]. This family is thought to be comprised of a zinc-dependent group of proteases, which play vital roles in a variety of normal and pathological conditions [2–7]. ADAMTS family was first identified in 1997 [8]. Since that time the ADAMTS family has grown to include 19 members, which are involved in a plethora of diseases ranging from arthritis to coagulation disorders [1, 9–19]. The ADAMTS family is divided into four subgroups based on their structural and functional similarities [8, 20–22]. ADAMTS-17 and -19, ADAMTS-16 and -18, ADAMTS-7 and -12, and ADAMTS-6 and -10 compose the structurally related ADAMTS pairs division. ADAMTS-12 was first reported by Cal et al. in 2001 [23]. It has been well established that the ADAMTS-12 gene is expressed in the musculoskeletal system (skeletal muscles, cartilage, and tendons) and in the fetal lung [1, 23, 24]. There has been increasing evidence pertaining to the pathological relevance of this enzyme. For instance, ADAMTS-12 has been proven to be associated with the pathogenesis of arthritis [8–16], intervertebral disc degeneration [25, 26], inflammation [27, 28], and invasion and metastasis of tumor [29–31]. Although ADAMTS-7 and -12 share similar structure and domain organization, ADAMTS-7 and -12 may have different pathological effects or be responsible for different phases in a disease [26, 32]. For instance, ADAMTS-7 mRNA is found to be significantly increased in the cartilage of patients with rheumatoid arthritis (RA) and only slightly increased in the cartilage of patients with osteoarthritis (OA), whereas ADAMTS-12 mRNA is significantly increased in both OA and RA cartilage [10, 32]. Moreover, in intervertebral disc degenerative (IVD) rat model, the level of ADAMTS-7 was significantly increased in the early phase while the level of ADAMTS-12 was increased in the latter phase [26]. In this review, we will discuss the structure and perspective of ADAMTS-12 and focus on its roles and the underlying mechanisms in arthritis (especially osteoarthritis) and other diseases, including inflammation, tumorigenesis and antiangiogenesis, intervertebral disc degenerative disease, schizophrenia, gonad differentiation, trophoblast invasion, and pediatric stroke (Figure 1). In addition, we used ADAMTS12 and ADAMTS-12 as keywords to search articles through Pubmed, Google Scholar, and Web of Science.

## 2. Structural Organization of ADAMTS-12

**Figure 2:**
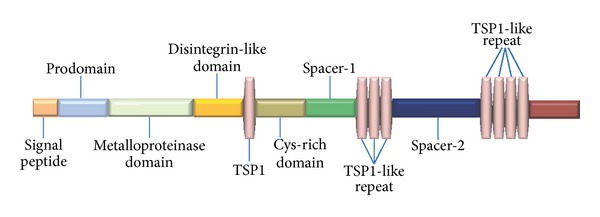
Domain structure and organization of ADAMTS-12. ADAMTS-12 N-terminal consists of a signal peptide, prodomain, and metalloprotease domain. The ADAMTS-12 C-terminal contains a disintegrin domain, the first thrombospondin type 1 repeat (TSP1), Cys-rich domain, and 7 additional TSP1 repeats interspaced with two spacer domains. The second spacer domain is also a mucin-like domain.

The structure of ADAMTS-12 includes a signal peptide, a prodomain, a catalytic domain, a disintegrin-like domain, a central thrombospondin I (TS) repeat, a cysteine-rich domain, two spacer regions, and C-terminal TSP-like repeat region (Figure 2) [33, 34]. It was believed that the C-terminal tail of ADAMTS-12 might be important for the ability of ADAMTS-12 binding to the components of extracellular matrix (ECM). Indeed, it was found that the four TSP-like repeat regions of ADAMTS-12 directly bound to the EGF-like domain of cartilage oligomeric matrix protein (COMP) [1]. It was also reported that the C-terminal four TSP-like repeat inhibited chondrocyte differentiation [35]. To determine the effects of each functional domain of ADAMTS-12 on chondrocyte differentiation, numerous deleted mutants were generated, such as the removal of C-terminal four thrombospondin motifs known to bind COMP, the removal of a spacer-2 domain, the deletion of three thrombospondin motifs, and the deletion of a spacer-1 domain. All of the above mentioned deletions did not change the cell surface localization of the protein in chondrocytes. However, the deletion of the cysteine-rich domain released the enzyme from cell surface into the medium. These data indicated that the cysteine-rich domain of ADAMTS-12 was required for its binding to ECM and cell surface appearance in chondrocytes [35]. In addition, the antiangiogenesis properties of ADAMTS-12 did not rely on the catalytic domain, but, more likely, on its TSP-1 domains [30]. In contrast, the catalytic activity was implicated in the protective effect against allergen-induced inflammation [28].

## 3. Biological Functions of ADAMTS-12

### 3.1. Arthritis

Arthritis is the leading cause of physical disability, health care usage, and impaired quality of life [36–38]. The impact of arthritic conditions is expected to grow as the elderly population increases in the coming decades [39, 40]. Osteoarthritis (OA) and rheumatoid arthritis (RA) are the most common categories of arthritis [8, 41–43]. OA is associated with age-related loss of homeostatic balance between degradation and repair mechanisms in articular cartilage, which is characterized by synovitis, cartilage degeneration, and osteophyte formation [44–47]. OA is a multifactorial disorder which can be affected by genetic alternation, inflammation, mechanical stress, obesity, and aging as well as biochemical, molecular, and enzymatic factors [48–51]. RA is a chronic systemic inflammatory disease that is characterized by the inflammatory proliferation of synovial tissues, leading to cartilage and bone damage and eventual disability [52–54]. Both genetic and environmental factors, as well as aberrant immune responses, play a key role in the pathogenesis of RA [55, 56]. The dysregulation in both OA and RA induces senescence, differentiation, proliferation, and cell death in joint cells through gene and/or protein expression networks that switch from anabolic to catabolic outcomes [48]. Recent evidence has demonstrated the significance of proteases, in the pathological processes of arthritis [57–59]. The destruction of the extracellular matrix of articular cartilage and bone in arthritic joints is thought to be mediated by excessive proteolytic activity [60]. In accordance with this concept, recent studies show that ADAMTS-12 also plays a critical role in the pathological process of arthritis [8, 32, 35, 43, 61].

### 3.2. In Osteoarthritis

Osteoarthritis is an age-related or posttraumatic degenerative disease of the joint that is characterized by loss of articular cartilage, chondrocyte proliferation and hypertrophic differentiation, subchondral bone remodeling, inflammation, and finally osteophyte formation [62, 63]. There is a strong relevance between cartilage degradation and osteoarthritis [64–66]. In that case, anything that can affect cartilage may play a role in the initiation and progression of osteoarthritis [63].

The finding that ADAMTS-12 was isolated as a binding metalloproteinase of COMP [1], an extracellular matrix highly expressed in cartilage [8, 32, 67–71], promoted us to determine the role of ADAMTS-12 in cartilage development and degeneration. It was reported that ADAMTS-12, functioning as a downstream molecule of parathyroid hormone related peptide (PTHrP) signaling, negatively regulated chondrogenesis [35]. ADAMTS-12 was prominently expressed in proliferating and prehypertrophic chondrocytes within the wild-type embryonic growth plate, whereas ADAMTS-12 was hardly detected in PTHrP(−/−) embryos. In addition, within the ADAMTS-12 overexpressing cells, the expressions of both early (collagen II and Sox9) and late (collagen X) marker genes for chondrogenesis were all repressed. In contrast, PTHrP expression was enhanced. Furthermore, PTHrP signaling was nearly abolished when ADAMTS-12 was repressed, and PTHrP action was largely restored when ADAMTS-12 was reexpressed. Notably, the negative regulation of chondrocyte differentiation by ADAMTS-12 depended on its enzymatic activity since its point mutant lacking enzymatic activity completely lost this activity [35].

The expression of ADAMTS-12 in the course of chondrogenesis was regulated by c-Maf transcription factor [72]. c-Maf, the cellular homologue of the avian viral oncogene* c-Maf*, was a member of the basic leucine zipper (bZIP) superfamily [73–75]. c-Maf proteins bind to a specific DNA sequences termed the “*Maf recognition element*” (*MARE*) through the basic DNA recognition domain [76]. In addition, c-Maf was overexpressed during embryonic development and postnatal growth in hypertrophic chondrocytes as well as in the osteoarthritic chondrocytes, which was similar to the expression pattern of that of ADAMTS-12 [73–75]. c-Maf and ADAMTS-12 were coexpressed in the process of* in vitro* chondrogenesis. Furthermore, c-Maf transcriptional factor directly binds to the proximal MARE sequences of ADAMTS-12 at position -61* in vitro* [72].

Interestingly, ADAMTS-12 was found to function as the downstream target of parathyroid hormone-like hormone/parathyroid hormone related peptide (PTHLH/PTHrP) in chondrocytes, and both PTHLH and ADAMTS-12 were all downregulated along with impaired chondrogenic differentiation within brachydactyly type E (BDE) [77]. BDE is an autosomal-dominant disease characterized by bilateral manifestation of shortened metacarpals, metatarsals, and/or phalanges [78–80]. Additionally, PTHLH and both of its targets, ADAMTS-7 and -12, were downregulated in the fibroblasts of BDE patients. Furthermore, the expressions of chondrogenic markers (aggrecan, collagen II, collagen X, and Indian hedgehog (IHH)) in chondrogenically differentiated BDE were all significantly altered. These findings suggested that that ADAMTS-12 and ADAMTS-7 are required for limb development.

**Figure 3:**
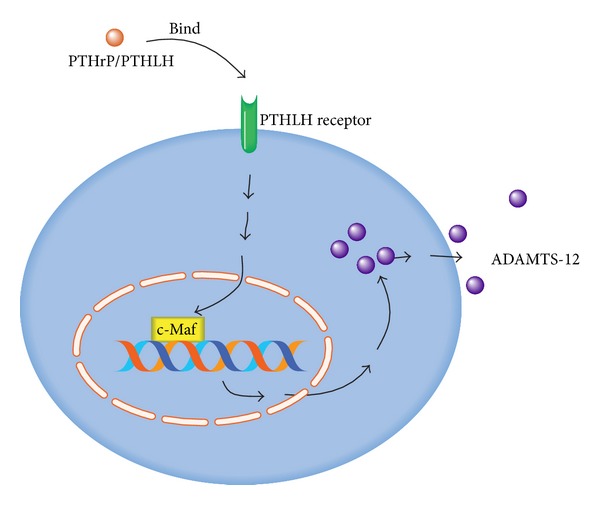
Regulation of ADAMTS-12 by PTHrP signaling in chondrocyte differentiation. ADAMTS-12 acts as a downstream molecule of PTHrP/PTHLH signaling and is regulated by c-Maf transcription factor in the course of chondrogenesis. Abbreviations: PTHrP/PTHLH: parathyroid hormone-like hormone; ADAMTS: a disintegrin and metalloproteinase with thrombospondin motifs.

Collectively, ADAMTS-12, whose expression was regulated by c-Maf transcription factor, acted as a downstream molecule of PTHrP signaling, negatively regulated chondrocyte differentiation, and hypertrophy (Figure 3). In addition, proper expression of ADAMTS-12 is required for normal cartilage development and its dysregulation may lead to pathologies with defects in the musculoskeletal system, such as BDE. Abnormal level and/or function of ADAMTS-12 may induce dysregulation of cartilage, which finally leads to arthritis.

ADAMTS-12's involvements in osteoarthritis is most probably due to its association and degradation of cartilage oligomeric matrix protein (COMP) [1, 8, 32, 67–71]. COMP, a prominent noncollagenous component of cartilage, accounts for approximately 1% of the wet weight of the tissue. The function of COMP may be involved in stabilizing cartilage ECM via specific interactions with matrix components such as collagen types II and IX, aggrecan, and fibronectin [81–83]. COMP also has a role in mediating chondrocyte attachment via an integrin receptor [84–87]. It was reported that mutations in the type III or C-terminal globular domain of the human COMP gene have been linked to the development of pseudoachondroplasia and multiple epiphyseal dysplasia, which were autosomal-dominant forms of short-limb dwarfism [88, 89]. COMP fragments have been detected in the diseased cartilage, synovial fluid, and serum of patients with knee injuries [90]. The level of ADAMTS-12 was significantly increased in the cartilage and synovium of patients with RA or OA [9, 10]. Both the* in vitro* glutathione S-transferase (GST) pull-down assay and coimmunoprecipitation assay demonstrated that ADAMTS-12 could bind to COMP directly [1]. Importantly, the size of COMP fragments generated by ADAMTS-12 was similar to those of COMP-degraded fragments seen in arthritic patients [43]. In addition, antibodies against ADAMTS-12 dramatically inhibited tumor necrosis factor-induced (TNF-induced) and interleukin-1*β*-induced (IL-1*β*-induced) COMP degradation in cartilage explants [43]. Suppression of ADAMTS-12 expression using the small interfering RNA silencing approach in human chondrocytes also markedly prevented COMP degradation. In addition, ADAMTS-7 and -12 were colocalized with COMP both in the cytoplasm and on the surface of human chondrocytes [25, 35]. While ADAMTS-7 was unable to digest aggrecan [91], ADAMTS-12, with a similar structure, did have the ability to digest aggrecan [5].

**Figure 4:**
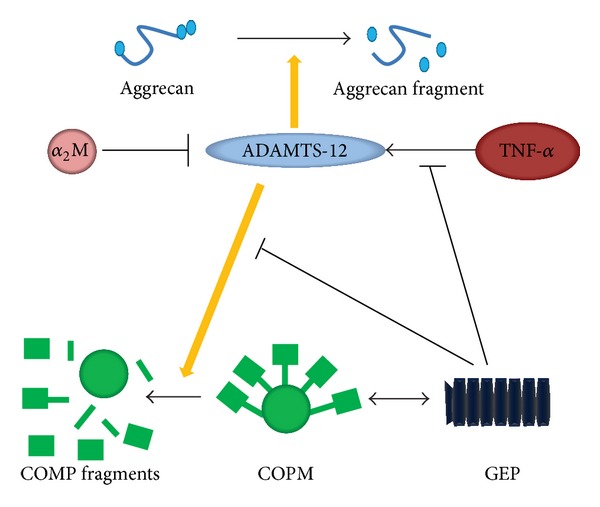
The interplay network in the degradation of extracellular matrix mediated by ADAMTS-12 in arthritis. ADAMTS-12 mediated proteolysis of aggrecan and COMP is positively modulated by TNF-*α* and inhibited by *α*_2_ M and GEP. Abbreviations: yellow arrows indicate a stimulatory effect; perpendicular lines indicate an inhibitory effect; *α*_2_ M: alpha-2-macroglobulin; ADAMTS: a disintegrin and metalloproteinase with thrombospondin motifs; TNF: tumor necrosis factor; COMP: cartilage oligomeric matrix protein; GEP: granulin-epithelin precursor.

It was reported that alpha-2-macroglobulin (*α*_2_ M) acted as a substrate for both ADAMTS-7 and ADAMTS-12 and efficiently protected COMP degradation by these enzymes by* in vitro* digestion assays [43]. In addition, granulin-epithelin precursor (GEP, a newly identified chondrogenic growth factor, which was also known as proepithelin, acrogranin, progranulin (PGRN), and GP88/PC cell-derived growth factor) inhibited the action of ADAMTS-12 preventing COMP degradation via two distinct mechanisms [61, 92–97]. In detail, GEP inhibits the induction of ADAMTS-12 by inflammatory cytokines such as TNF-*α* and GEP is able to disrupt the association between ADAMTS-12 and COMP via a direct protein-to-protein interaction [8, 61]. It was approved that ADAMTS-12, COMP, aggrecan, GEP, *α*_2_ M, and TNF-*α* constitute an interplay and interaction network in mediating cartilage degradation in arthritis (Figure 4). TNF-*α* upregulates ADAMTS-12 leading to the degradation of COMP [43, 98, 99]. In addition, TGF-*β* was reported to significantly induce the ADAMTS-12 in human fetal fibroblasts [21].

This* in vitro* data provide insight into the critical role of ADAMTS-12 in the initiation and progression of osteoarthritis which needs to be further confirmed using* in vivo* genetically modified animal models. Interestingly, such* in vivo* models for ADAMTS-7, which shares similar domain organization and structure, have been generated, and the critical role of ADAMTS-7 in the pathogenesis of OA has been elucidated [58].

It was reported that ADAMTS-7 formed a positive feedback loop with TNF-*α* in the pathogenesis of osteoarthritis [58]. ADAMTS-7 expression was elevated during disease progression in the surgically induced rat OA model. Targeted overexpression of ADAMTS-7 in chondrocytes led to chondrodysplasia characterized by short-limbed dwarfism and a delay in endochondral ossification in “young mice” and a spontaneous OA-like phenotype in “aged” mice. In addition, overexpression of ADAMTS-7 led to exaggerated breakdown of cartilage and accelerated OA progression, while knockdown of ADAMTS-7 attenuated degradation of cartilage matrix and protected against OA development, in surgically induced destabilization of medial meniscus OA models [58, 100]. ADAMTS-7 upregulated TNF-*α* and metalloproteinases associated with OA; in addition, TNF-*α* induced ADAMTS-7 through NF-*κ*B signaling. These data indicated that ADAMTS-7 and TNF-*α* form a positive feedback loop in the regulation of cartilage degradation and OA progression [58, 101]. Aside from the fact that ADAMTS-12 shares a similar structure with ADAMTS-7, ADAMTS-12 may play a critical role in the OA development* in vivo* [10, 23, 58].

### 3.3. In Rheumatoid Arthritis

Rheumatoid arthritis (RA) is a chronic systemic inflammatory disease, characterized by inflammation of the synovial membrane and progressive destruction of the articular cartilage and bone [102, 103]. As we have previously stated, ADAMTS-12 is an inflammation-related protein, which can digest COMP; it was reported that ADAMTS-12 might play a role in RA [104, 105]. ADAMTS-12 was recently identified as a susceptible gene for RA [104]. Three single nucleotide polymorphisms (SNPs) (*rs1364044, intron C/T*;* rs10461703, intron C/T*;* rs25754, missense Thr1495Ile*) of ADAMTS12 were genotyped by using a direct sequencing method in 303 RA patients and 495 control subjects. Multiple logistic regression models, SNPStats and SNPAnalyzer Pro programs, and Bonferroni's correction (pc) were used and found that the genotype frequency of* rs10461703* was associated with the RA development. However, no significant correlation was observed between the three tested SNPs and RA patients with regard to their clinical features [104].

Clinically, women are more susceptible to RA than men [105–107]. ADAMTS-12 was reported to associate with sex-specific disparities in RA resulting in cartilage degradation [105]. The involvement of sex chromosomes in RA and the relationship between animal models of RA and gender have been reported by several laboratories [108–114]. In addition, it was suggested that collagen antibody-induced arthritis (CAIA) in congenic mice can be used to identify homologous RA associated molecular pathways. It was reported that BALB/c.DBA/2-Pgia8- (*C.D2-Pgia8-*) congenic mice carry a sex-affected arthritis-suppressive genomic interval which was associated with PGIA [112]. PGIA mice were used in order to investigate CAIA susceptibility [105]. It was revealed that CAIA severity in Pgia8-congenic females was 20% higher than in BALB/c females. However, the inflammation severity of the Pgia8-congenic males was 30–45% lower than wild-type BALB/c males, indicating that the genetic anti-inflammatory effect was specific towards male. Criteria were set as a 1.5-fold change threshold in the CAIA severity change and three genes were found including collagen triple-helix repeat-containing 1 (*Cthrc1*), C1q and TNF-related protein 3 (*C1qtnf3*), and* ADAMTS-12*, which were all associated with both gender and arthritis [105]. In addition, these three genes were highly involved in the severity of arthritis with *r* = 0.91, 0.89, and 0.87, respectively; thus there is a strong correlation among these 3 genes. It was also demonstrated that the Cthrc1 protein positively regulated the PCP-Wnt pathway altering chondrocyte maturation and cartilage formation [115–117].

Collectively, these genetic and* in vitro* studies indicate that ADAMTS-12 plays an important role in the pathogenesis of arthritis and may be a potential target for developing new treatments for arthritis. ADAMTS-12-deficient mice model with arthritis are critical for verifying its role in the course of both osteoarthritis and rheumatoid arthritis* in vivo*.

## 4. Other Conditions and Diseases

### 4.1. Inflammation

ADAMTS-12 has also been reported to be a critical mediator in inflammation, especially allergic inflammation [24, 27, 28]. ADAMTS-12 has been identified as one asthma-associated gene in the human genome screening program [24]. Asthma is characterized by a specific pattern of inflammation and airway/bronchial hyperresponsiveness (AHR/BHR) [118, 119]. Fine mapping and positional candidate studies were performed in the Hutterites (South Dakota) and an outbred case-control sample from Germany [24]. Genotyping on chromosome 5p showed that SNPs in ADAMTS-12 were primarily associated with bronchial hyperresponsiveness (BHR) in the Hutterites. In addition, variation of ADAMTS-12 was also associated with asthma in the outbred Germans. Furthermore, there was a significant difference between asthma cases and controls in the frequencies of  long range haplotypes composed of SNPs at ADAMTS-12 [24].

Recently, asthma OVA and HDM model were generated in the ADAMTS-12-deficient mice to determine the role of ADAMTS-12 in this disease [28]. It was found that ADAMTS-12 deficiency could exacerbate an allergen-induced inflammatory and airway response by inducing Th2 inflammation [28]. ADAMTS-12-deficient mice models displayed a more severe phenotype of allergen-induced AHR when compared to their corresponding WT littermates by histological analysis. In addition, the markers of Th2-skewed inflammatory response, such as Ag-specific IgE and Ag-specific IgG1, were all significantly increased in allergen-induced mice. And there appeared to be no difference between the groups when evaluating the Th1-associated marker, IgG2a. The cytokine levels of IL-4 and IL-13 (typical Th2 cytokines) were highly increased in ADAMTS-12-deficient mice. On the other hand the levels of IL-10 and IFN-*γ* (Th1 cytokines) did not exhibit significant changes. Furthermore, the levels of RANTES (CCL5) (a major eosinophil chemoattractant [120]), IL-5 (known to regulate eosinophil differentiation and survival [121]), and IL-33 (known to further contribute to exacerbated eosinophil [122, 123]) were all increased and coordinate with the increased amount of eosinophilia. IL-33 could most notably increase mast cell recruitment through ST2 receptor activation. The levels of both the mast cells and ST2 receptor were higher within the lungs of allergen-induced ADAMTS-12-deficient mice [124, 125].

In addition to its involvements in allergic inflammation, ADAMTS-12 was found to be required in normal inflammation [27]. To elucidate the role of ADAMTS-12 in normal inflammation, several* in vivo* murine models have been studied. ADAMTS-12-deficient mice were used to create the animal models for colitis, endotoxic sepsis, and pancreatitis [27]. ADAMTS-12-deficient mice exhibited more severe inflammation. They also experienced a delay in the recovery process from these challenges by comparison with their wild-type littermates. The ADAMTS-12-deficient tissues showed a significant increase in several inflammatory markers in both RNA and protein levels after analysis. In addition, all ADAMTS-12-deficient mice models exhibited severe inflammatory symptoms accompanied by neutrophilia in the affected tissues. ADAMTS-12 exhibited a protective role in inflammatory pathogenesis [27]. ADAMTS-12 achieved this by inducing the apoptosis of neutrophils. Furthermore, hemopexin and cytokines such as granulocyte-colony stimulating factor (GCSF) and IL-6 were increased in the ADAMTS-12-deficient mice. Notably, hemopexin and GCSF had the ability to promote duration and inhibit the apoptosis of neutrophils [126–128]. IL-6 was also reported to function as compensation for limiting neutrophils recruitment [129].

Above all, ADAMTS-12 has a genetic linkage with asthma and a deficiency in ADAMTS-12 leads to defects in both the normal and hyperresponsive inflammatory response. It may act as an important mediator maintaining the immune balance in both biological and pathological process. Clearly, the molecular events and mechanisms underlying ADAMTS-12-mediated regulation of inflammation warrant further investigations.

### 4.2. IVD Degeneration

Within intervertebral disc (IVD) degeneration the subchondral endplate condenses to a denser structure. This process may negatively influence both the diffusion and nutrition of the IVD, which can lead to a more sever phenotype. It was reported that the expressions of ADAMTS-7 and ADAMTS-12 in the endplate cells isolated from patients with degenerative disc disease were significantly increased [25]. A rat caudal intervertebral disc model was used to examine the expression profiling of ADAMTS-7 and -12 in the course of the degeneration of intervertebral disc [26]. ADAMTS-7 and ADAMTS-12 were detected in the NPs and their expressions were slightly changed within the first 18 hours after loading. After that window of time both ADAMTS-7 and -12 increased significantly and coordinated with the dramatic increase of COMP. Within a recent load-induced IVD degenerative rat model, the expression of ADAMTS-7 peaked on the first day and ADAMTS-12 expression reached peak level on the 7th day. However, on 7th day, the level of ADAMTS-12 was much higher than that of ADAMTS-7. This data suggests that ADAMTS-7 and ADAMTS-12 may contribute to the early and late degeneration stage of IVD, respectively [26].

### 4.3. Tumor Suppression and Angiogenesis Inhibition

ADAMTS-12 was found to be expressed in the fibroblasts adjacent to the tumor cells in the colorectal cancer specimens from human patients [31]. In addition, the expression of ADAMTS-12 was not associated with the tumor origin, nor gender or age, but depended on the differentiation of the cancer cells. Well-differentiated cells exhibited a higher staining score while poorly differentiated cells displayed negative staining. Moreover, the ADAMTS-12 expression in cancer stroma of patients with lymph node metastasis was greater than that within the patients without lymphatic metastasis. Therefore, ADAMTS-12 may be a promising prognostic indicator for colorectal cancer [31].

The actual secretion of ADAMTS-12 was lost by the epigenetic silencing in tumor cells [130]. This silencing occurs in a compensatory manner within induced stromal cells. The ADAMTS-12 gene promoter was hypermethylated in colorectal carcinomas and colon cancer cell lines resulting in the silence of ADAMTS-12 expression [29]. In addition, it was reported that ADAMTS-12 was expressed in fibroblastic-like cells surrounding malignant cells [29], and it was confirmed by histological staining from clinic [31]. Moreover, well and moderately differentiated carcinomas showed a strong immunoreactivity for ADAMTS-12 whereas poorly differentiated cells showed weak staining. The expression of ADAMTS-12 was doubled when fibroblasts coculture with colon cancer cells compared with fibroblasts cultures alone. It has been documented that the tumor cell growth rate was significantly decreased when fibroblasts coculture with colon cancer cells, as compared to colon cancer cell cultures alone. Furthermore, the apoptotic cancer cell population of cocultured cells with colon fibroblasts was much higher than that of tumor cell cultures alone. It was reported that fibroblasts-expressed ADAMTS-12 acted as a compensatory strategy for the epigenetic silencing of this gene within malignant cells.

ADAMTS-12 was reported to exact an tumorigenesis and angiogenesis-inhibitory effect [5, 29–31, 131]. ADAMTS-12 was found to play a protective role in angiogenesis and cancer progression via using ADAMTS-12-deficient-mice models [30]. Malignant keratinocyte (PDVA cell line) transplantation demonstrated that tumor vascularization and invasion were increased in ADAMTS-12-deficient-mice. In addition, this observation was also confirmed by a computerized image analysis [132]. The aortic explants assay and basic fibroblast growth factor (bFGF) injection indicated that ADAMTS-12 was a negative regulator of angiogenesis [133]. Additionally, the overexpression of ADAMTS-12 in breast carcinoma (MCF7 cell line) resulting in reduced new vessel formation exhibited an inhibitory effect in angiogenesis as well [30]. ADAMTS-12 was also found to abolish vascular endothelial growth factor- (VEGF-) induced tubule formation in the bovine aortic endothelial (BAE) cells [5]. Furthermore ADAMTS-12 inhibited tumor growth* in vivo* as well, since injection of A549 cells overexpressing ADAMTS-12 into the immunodeficient-SCID-mice showed a reduced tumor growth [5].

ADAMTS-12 was reported to play its tumorigenesis role by modulating the Ras-dependent ERK signaling pathway [5]. There was a notable lower level of phosphorylated ERK in the Madin-Darby canine kidney (MDCK) cells expressing ADAMTS-12 than those lacking ADAMTS-12 after being stimulated by hepatocyte growth factor (HGF) [5]. In addition, ADAMTS-12 may function as a tumor suppressor through limiting the proliferation of tumor cells [29]. Interestingly, the catalytic activity of ADAMTS-12 appeared to be dispensable for its angiogenic-inhibitory function, since the mutations in the catalytic domain inactivated its enzymatic activity but did not impair the antiangiogenic effect [30].

New findings have illustrated that ADAMTS-12 could elicit tumorigenesis effects when it interacts with fibulin-2 in breast cancer [131]. Fibulin-2 belongs to fibulins, which are components of basement membranes and elastic matrix fibers in connective tissue and also play a role in tumorigenesis [134–136]. Both* in vitro* and* in vivo* data have shown that ADAMTS-12 itself could exhibit protumor effect while interaction of ADAMTS-12 and fibulin-2 could exhibit tumorigenesis effect [131]. Furthermore, clinically detection of both proteins in breast cancer patients predicted a good prognosis [131].

Taken together, both* in vivo* and* in vitro* experiments demonstrate that ADAMTS-12 expression associates with the differentiation of the cancer cells and has tumorigenesis and antiangiogenic effects. Thus, ADAMTS-12 may provide a new molecular target for treating various types of cancer in addition to the potential of being employed as a prognostic indicator in tumor malignant grade and metastasis.

### 4.4. Schizophrenia

Members of ADAMTS family have been implicated in neurodegenerative and neuroinflammatory diseases [137–139]. In addition, some metalloproteinases, including MMP-9 and ADAMTSL3, were found to be implicated in schizophrenia [140, 141]. Recently ADAMTS-12 was also reported to have an association with schizophrenia [142]. The ADAMTS-12 gene had been shown to be a functional and positional candidate in the susceptibility of schizophrenia by mutation analysis in Puerto Rican patients of Spanish descent. Moreover, the intronic variant* rs256792* and the two-SNP haplotype* rs256603–rs256792* were closely associated with the disorder, although the function of single nucleotide polymorphisms remains largely unknown.

### 4.5. Gonad Differentiation

ADAMTS-12 was recently isolated as a gene involved in the gonadal development [143]. Briefly, 110 genes were selected for evaluation of their expression during chicken gonad differentiation, and ADAMTS-12 showed higher level of expression within the testis and was increased during gonadal development [143]. Furthermore, whole mount* in situ* hybridization assay reveled that ADAMTS-12 was expressed in the testis on day 7, the time of histological differentiation of the gonads. In addition, ADAMTS-12 was located in the developing testicular cords. A previous study reported that ADAMTS-12 acted as an inhibitor of SOX9 expression during* in vitro* chondrocyte differentiation [143] and the reverse expression profiling between ADAMTS-12 and Sox9 in the course of chicken gonad differentiation indicating that ADAMTS12 may be a novel regulator of gonad differentiation through modulating SOX9 transcription factor. In addition, it was reported that ADAMTS-12 might be an early marker of male gonad differentiation [143].

### 4.6. Trophoblastic Cell Invasion

In addition to its potential role in male gonad differentiation, ADAMTS-12 was also found to be involved in the trophoblastic cell invasion [144, 145]. ADAMTS-12 was found to be detectable in samples of human placental tissue from the first trimester [145]. ADAMTS-12 altered the cell-extracellular matrix (ECM) interactions leading to the reduction of cell adhesion capability and promotion of cell invasion by measuring the first trimester trophoblastic cells. Expression of ADAMTS-12 was significantly higher in cultures of invasive extravillous cytotrophoblast (EVT) than in poorly invasive JEG-3 choriocarcinoma cells. ADAMTS-12 was found to promote cell invasion using both overexpression and siRNA knockdown assays. In addition, it was reported that gonadotropin-releasing hormone- (GnRH-) I and II increased ADAMTS-12's expression in EVT within a time- and concentration-dependent manner [144] and transforming growth factor-*β*1 (TGF-*β*1) promoted cytotrophoblast invasion* in vitro* while interleukin-1*β* (IL-1*β*) restrained cytotrophoblast invasion* in vitro* [145]. In addition, ADAMTS-12 reduced cell-EMC adhesion by regulating the *α*v*β*3 integrin heterodimer [145]. Furthermore, the ability of ADAMTS-12 to promote invasion largely depended on the disintegrin-like domain and ancillary domains [144].

### 4.7. Pediatric Stroke

It was reported that there was a relationship between the ADAMTS family and pediatric stroke [146]. Pediatric stroke was a heterogeneous disorder associated with significant morbidity and mortality. Although SNPs residing in ADAMTS12 were strongly associated with pediatric stroke [146], the mechanisms through which the genetic variants in ADAMTS genes lead to pediatric stroke still remain unknown.

## 5. Summary and Perspective

ADAMTS-12 protease is involved in the processes of chondrocyte differentiation and cartilage development. The dysregulation of ADAMTS-12 is associated with musculoskeletal degenerative diseases, including arthritis and IVD degeneration, which probably resulted from its degradation of COMP, aggrecan, and other unidentified matrix proteins and its role in inflammation. In addition, ADAMTS-12 functions as a protective mediator in both normal and hyperresponsive inflammation by inducing neutrophil apoptosis and interfering with the T response, respectively. Furthermore, ADAMTS-12 is found to exhibit tumorigenesis and angiogenic-inhibitory effect in certain cancer types and ADAMTS-12 has the potential to be used as an indicator of cancer prognosis. ADAMTS-12 also promotes the invasive ability of trophoblastic cells and acts as an early marker of male gonad differentiation in chicken.

Although growing evidence indicates that ADAMTS-12 is a metalloproteinase with multiple functions, the exact expression profiling, regulation, and function of ADAMTS-12 in various pathophysiological processes, especially the signaling pathways and molecular events involved, remain to be delineated. Moreover, the function of each domain should be taken into account since it has been reported that thrombospondin type 1 repeats interact with MMP-2, whose role in osteoarthritis is currently emerging [147]. By determining the functional domain of ADAMTS-12 in osteoarthritis, we can develop a more efficient inhibitor to rescue this disease. In addition, the protein partner of ADAMTS-12 in the progression of osteoarthritis is also a big challenge to us.

Although global knockout mice of ADAMTS-12 are viable, timely controlled (i.e., inducible), tissue-specific knockout and/or transgenic mice are needed for precisely dissecting the role of ADAMTS-12 in the development of various tissues. In addition, these mouse strains are especially useful for generating various kinds of diseases models. For instance, in order to figure out the potential effect of ADAMTS-12 in cartilage development and the initiation and/or progression of OA, cartilage specific deletion of ADAMTS-12 in the adult mice is required for generating surgically induced OA models.

## Abbreviations

ECM: Extracellular matrix
COMP: Cartilage oligomeric matrix protein
ADAMTS: A disintegrin and metalloproteinase with thrombospondin motifs
MMP: Matrix metalloproteinase
IVD: Intervertebral disc
PTHrP/PTHLH: Parathyroid hormone-like hormone
MARE: Maf recognition element
BDE: Brachydactyly type E
SNPs: Single nucleotide polymorphisms
OA: Osteoarthritis
RA: Rheumatoid arthritis
CAIA: Collagen antibody-induced arthritis
GEP: Granulin-epithelin precursor
*α*_2_M: Alpha-2-macroglobulin
NP: Nucleus pulposus
EVT: Extravillous cytotrophoblast
GnRH: Gonadotropin-releasing hormone.
